# How potentially predictable are midlatitude ocean currents?

**DOI:** 10.1038/srep20153

**Published:** 2016-02-01

**Authors:** Masami Nonaka, Yoshikazu Sasai, Hideharu Sasaki, Bunmei Taguchi, Hisashi Nakamura

**Affiliations:** 1 Japan Agency for Marine-Earth Science and Technology, Yokohama, 236-0001, Japan; 2 Research Center for Advanced Science and Technology, University of Tokyo, Tokyo, 153-8904, Japan

## Abstract

Predictability of atmospheric variability is known to be limited owing to significant uncertainty that arises from intrinsic variability generated independently of external forcing and/or boundary conditions. Observed atmospheric variability is therefore regarded as just a single realization among different dynamical states that could occur. In contrast, subject to wind, thermal and fresh-water forcing at the surface, the ocean circulation has been considered to be rather deterministic under the prescribed atmospheric forcing, and it still remains unknown how uncertain the upper-ocean circulation variability is. This study evaluates how much uncertainty the oceanic interannual variability can potentially have, through multiple simulations with an eddy-resolving ocean general circulation model driven by the observed interannually-varying atmospheric forcing under slightly different conditions. These ensemble “hindcast” experiments have revealed substantial uncertainty due to intrinsic variability in the extratropical ocean circulation that limits potential predictability of its interannual variability, especially along the strong western boundary currents (WBCs) in mid-latitudes, including the Kuroshio and its eastward extention. The intrinsic variability also greatly limits potential predictability of meso-scale oceanic eddy activity. These findings suggest that multi-member ensemble simulations are essential for understanding and predicting variability in the WBCs, which are important for weather and climate variability and marine ecosystems.

Ocean gyre circulation within the upper 1000 meters or so is known to be driven mainly by surface wind stress^1^,^2^,^3^,^4^, and wind-forcing variability is thus believed to be the dominant source of interannual variability in the gyre circulation through oceanic dynamical adjustment^5^. Compared to its atmospheric counterpart, interannual variability in the ocean circulation has therefore been considered to be far more deterministic, and not much attention has been paid to its chaotic behavior that could arise intrinsically from oceanic internal dynamics.

Exceptions have recently been found in the western boundary currents (WBCs), including the Gulf Stream and the Kuroshio. These strong warm currents along the western boundaries of large ocean basins are known to be important for weather and climate variability^6^,^7^,^8^ and marine ecosystems^9^. These currents, especially their eastward jets extending off the continental east coasts, simulated in ocean models are shown to vary interannually even under the seasonally-varying wind forcing without any interannual variability^10^,^11^,^12^,^13^,^14^. For this type of model setting, the simulated interannual variability of the ocean currents should be regarded as intrinsic variability. As its atmospheric counterpart, it is generated internally through instability of the currents and nonlinearity of ocean eddies independently of any external forcing. Through those model simulations, however, one cannot quantitatively evaluate how much uncertainty the oceanic intrinsic interannual variability can bring to the entire interannual variability of the currents, due to the lack of the atmospheric-driven variability. As intrinsic variability is inherently unpredictable, potential predictability of the entire oceanic interannual variability is presumably low where the oceanic intrinsic variability dominates the atmospheric-driven variability. The entire interannual variability, however, cannot be estimated simply as the algebraic sum between the former and latter types of variability, owing to nonlinear nature of the former. Therefore an assessment of the potential predictability of the ocean current variability is not necessarily straightforward. It is required to investigate how large the oceanic intrinsic variability is under realistic interannually-varying atmospheric forcing.

For our assessment of the potential predictability, we first focus on the variability of a prominent eastward jet of the Kuroshio Extension (KE). As revealed by satellite observations, the KE jet wobbles on decadal scales between stable and unstable states in its path^15^, which is caused by westward propagation of oceanic Rossby waves forced under large-scale wind variability over the central/eastern North Pacific^15^. This wind-forced KE variability is reproduced in a long-term integration of an eddy-resolving ocean general circulation model (OGCM)^13^,^16^. Meanwhile, a two-layer ocean model with coastlines mimicking the North Pacific^11^ and a realistic eddy-resolving OGCM^13^ also simulates decadal-scale modulations of the KE jet occurring under the seasonal wind forcing with no interannual variability. In the present study, we analyze both the intrinsic and atmospheric-driven variability of the KE jet in three-member ensemble OGCM experiments driven by interannually-varying atmospheric forcing to elucidate the relations between the two types of ocean variability.

## Results

**Figure 1:**
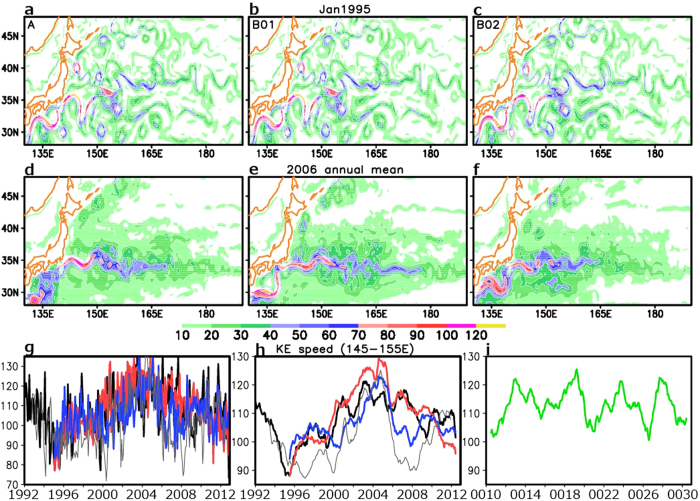
100-m depth current speed in hindcast experiments. (**a–f)** Maps for the ensemble members A (**a,d**), B01 (**b,e**) and B02 (**c,f**) in shades. (**a–c)** Monthly means for January 1995. (**d–f)** Annual means for 2006. (**g–i**) Time series of the Kuroshio Extension jet speed averaged over 145–155 °E (**g**). Unit for every panel is cm s^−1^. In (**h**) 13-month running mean is applied to the time series in (**g**). Black, red, and, blue curves are for the ensemble members A, B01 and B02, respectively. Thin grey curve is for that derived from the satellite observed SSH. (**i**) Same as (**h**) but for the climatological integration. All plots are generated with GrADS.

As evident in Fig. 1a–c, the model-simulated western North Pacific currents are almost identical among the three members during the first month (January 1995) of the model integrations, owing to very slight differences in the initial conditions among the members (see Methods). Though driven by the identical atmospheric forcing, however, the simulated KE jet evolves quite differently in time among the three members due to oceanic intrinsic variability. In the annual-mean fields, for example, the KE jet in 2006 (Fig. 1d–f) differs notably among the members in its strength and zonal extent. Specifically, in the members A, B01, and B02, 40 cm s^−1^ contours extend as far eastward as 170 °E, 178 °E and 165 °E, respectively. The annual average was taken to suppress the contributions from short-lived meso-scale eddies that are generated spontaneously through dynamical instability of the KE jet and thus behave differently, by nature, among the members. The figures therefore suggest that the internal ocean dynamics limits the potential predictability of the KE jet.

The different evolution of the KE jet among the ensemble members is highlighted in the time series of the KE jet speed averaged longitudinally between 145 °E and155 °E. The time series based on the monthly-mean field (Fig. 1g) are dominated by high-frequency variability associated with eddies, but they are still almost identical in January 1995. Within a few months, however, the simulated KE speed begins to diverge as eddies develop differently among the members. The inter-member diversity of the KE speed is evident also in its 13-month running-mean time series (Fig. 1h), in which the eddy contribution has mostly been suppressed to highlight interannual variability of the jet. The root-mean-square (rms) difference (excluding the mean bias) among the three members from 1995 to 2012 is 6.86 cm s^−1^, which is comparable to the interannual standard deviation of the ensemble-mean speed over the members (7.36 cm s^−1^). The latter variability is driven mainly by wind variations, since the intrinsic ocean variability is largely cancelled out in the ensemble mean. The results suggest that both the unpredictable intrinsic variability and the potentially predictable wind-driven variability contribute comparably to the interannual variability in the KE jet speed. Furthermore, the rms difference among the three members is slightly larger than the interannual standard deviation of the KE jet speed (5.43 cm s^−1^) simulated in the climatological integration (Fig. 1i, see Methods), which purely represents oceanic intrinsic interannual variability. Due to the contributions from intrinsic variability, the simulated KE jet speed evolves differently among the individual members in strengthening and weakening at different timings. Among the three, only the B02 (blue curve in Fig. 1h) simulates the KE variability rather accordingly with the observed variability (grey thin curve). The inter-member diversity shown in our ensemble simulations urges caution that comparing a single OGCM integration under observed atmospheric forcing with the observed evolution of ocean currents may be misleading, though often put into practice for evaluating model’s fidelity in reproducing observed interannual variability.

On decadal scales, however, the KE evolution simulated in each of the three members (A, B01 and B02) seems similar to its observational counterpart, as the simulated jet speed minimizes around 1995 and maximizes around 2005 as observed. When low-pass filtering with 37-month running mean is applied to extract and highlight decadal-scale variability, the rms-difference in the KE jet speed among the three ensemble members (5.11 cm s^−1^) tends to be smaller than the corresponding standard deviation of the ensemble mean (6.16 cm s^−1^). This result implies that atmospheric variability may be more influential on decadal variability in the KE jet speed than on its interannual variability, as consistent with a pace-making effect of wind-forced large-scale ocean anomalies on internal variability^13^. The aforementioned estimates, however, are based on rather short time series, and specific magnitude of rms values may vary on decadal scales depending on regimes of the KE jet.

**Figure 2:**
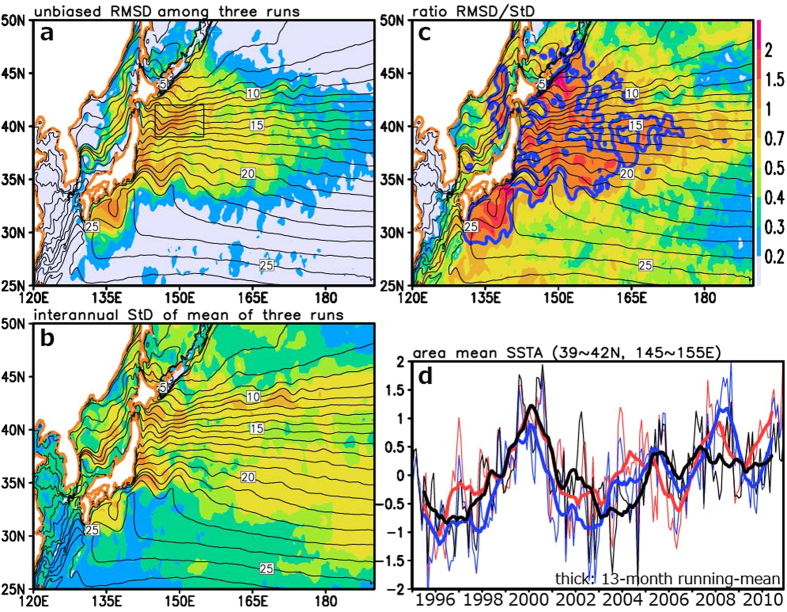
Uncertainty in sea-surface temperature (SST) fields. (**a)** Root-mean-square differences (RMSD) in SST anomalies (SSTAs) among the three ensemble members. (**b**) Standard deviations (StD) of the ensemble-mean SSTAs based on those three members. (**c**) Local ratio of the quantity in panel (**a**) to that in (**b**), with thick-blue contours for 1.0. (**d**) Time series of area-mean SSTAs averaged in the subarctic frontal zone (36–42 °N, 145–155 °E; rectangular in (**a**) based on the ensemble members A (black), B01 (red), and B02 (blue). Unit for the panels (**a**,**b,d)** is °C. 13-month running mean is applied to suppress the contributions from eddies in (**a–c)** and the thick curves in (**d)**. In (**a–c**) black contours indicate the mean SST. All plots are generated with GrADS.

The influences of intrinsic variability are found not only in the KE jet speed but also in temperature and salinity fields. The rms-difference in interannual anomalies in sea-surface temperature (SST) among the three members maximizes locally along the KE and the subarctic frontal zone to its north (Fig. 2a), where horizontal SST gradient is sharp and variability of the ocean currents generates strong SST variability^16^,^17^. Although SST anomalies forced by atmospheric variability are also large in the KE and subarctic frontal zones east of Japan (Fig. 2b), the rms-difference due to intrinsic variability tends to be even greater (Fig. 2c). In the frontal zones, the signal-to-noise ratio for the predicted SST interannual variability is less than unity, indicative of limited potential predictability. Significant differences in SST anomalies can be found also in their averages within the subarctic frontal zone (Fig. 2d), where SST fluctuates vigorously on interannual to decadal scales and feedback to the atmospheric circulation has been suggested on regional^18^,^19^,^20^ through basin scales^21^,^22^,^23^,^24^. As shown in Fig. 2d, the area-mean SST difference reaches nearly 1K around 2004 and timings of maxima and minima are not quite coherent among the members, although dominant interannual to decadal variability is rather similar.

**Figure 3:**
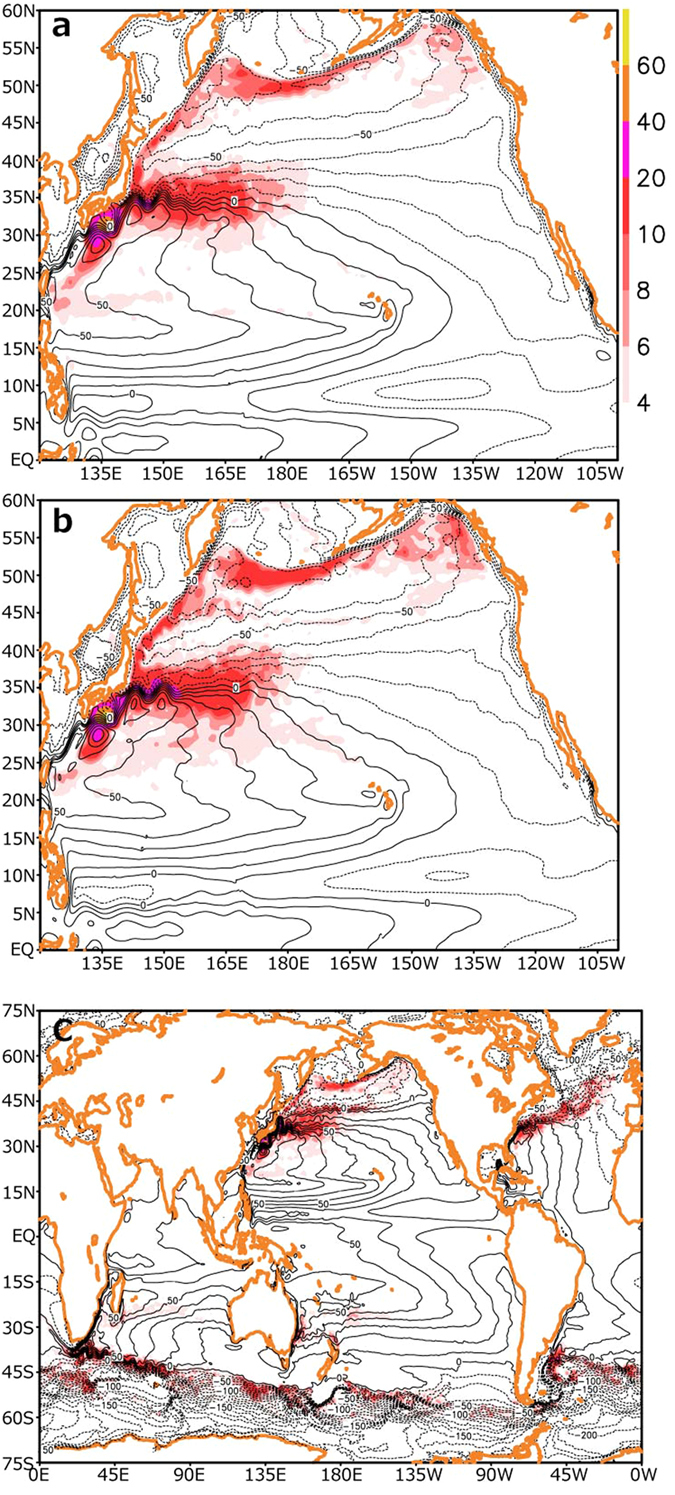
Uncertainty in sea-surface height (SSH) field. (**a)** Map of root-mean-square differences in 13-month running-mean SSHAs among the three ensemble members. Contours indicate the long-term mean SSH field with intervals of 10 cm. (**b**) Standard deviations of SSH for the climatological integration. (**c**) Same as (**b**) but for the quasi-global model integration. For every plot, unit is cm. All plots are generated with GrADS.

Significant influence of intrinsic variability is largely limited to the western North Pacific, whereas contrastingly in the tropics, oceanic variability is tightly coupled with atmospheric variability and could therefore be deterministic if forced by prescribed atmospheric forcing. As evident in Fig. 3a, rms-difference in interannual anomalies in sea-surface height (SSH) is diminished in the tropics south of 15 °N but much larger in mid-latitudes. Though overall similar to that of the rms-difference in SST variability, the rms-difference in SSH maximizes along the KE jet while that in SST along the subarctic frontal zone at 40 °N (Fig. 2a). Before plotting Fig. 3a, low-pass filtering has been applied so as to suppress signals of eddies. Eventually, high-frequency variability associated, for example, with tropical instability waves^25^ should therefore be suppressed in Fig. 3a.

The climatological integration simulates purely intrinsic variability measured as standard deviations in interannual SSH anomalies (Fig. 3b), whose geographical distribution and amplitude are very similar, though not identical, to their counterpart in the hindcast integrations (Fig. 3a). This result indicates that intrinsic interannual variability of the ocean is rather insensitive to interannually varying atmospheric forcing, and therefore the climatological integration may be used as a proxy of intrinsic variability simulated in hindcast integrations. As suggested in Fig. 3c, a climatological integration with a quasi-global eddy-resolving OGCM (see Methods) simulates intrinsic interannual variability, which is virtually absent over the tropical oceans but quite strong along the oceanic jets associated with the Antarctic Circumpolar Current and the warm WBCs, including the Agulhas, Brazil-Malvinas and East Australian Currents in the southern ocean, the Gulf Stream and Kuroshio, and their extension regions, consistent with the latest study with another eddy-resolving OGCM^26^. Since SSH gradients are particularly strong across these jets, changes in their speed and position arising from their intrinsic interannual variability tend to yield large SSH anomalies.

**Figure 4:**
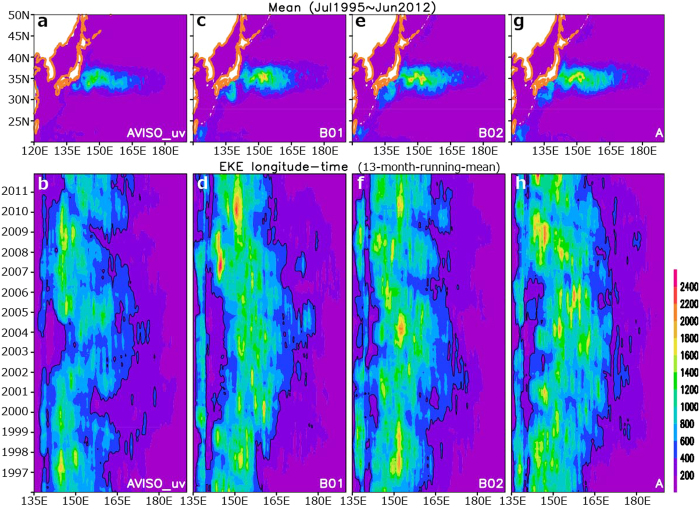
Eddy activity in the Kuroshio Extension region. Maps (**a,c,e,g**) and longitude-time sections of eddy kinetic energy (EKE) averaged over 32–37 °N (**b,d,f,h**). Unit is cm^2^ s^−2^. EKE is defined as (*u*’^2^ + *v*’^2^)/2, where *u*’ and *v*’ denote anomalies in the zonal and meridional current velocities, respectively, at the surface, derived from anomalous surface geostrophic velocities from satellite-measured SSHAs (**a,b**) and from the ensemble members B01 (**c,d**), B02 (**e,f**), and A (**g,h**). The eddy-associated anomalies have been obtained as high-pass-filtered deviations from the climatological seasonal march and 13-month running-mean anomalies superimposed. For panels (**b,d,f,h**) 13-month running mean is applied to EKE to highlight its interannual modulations. All plots are generated with GrADS.

Across these jets, thermal contrasts are also particularly strong, and therefore meso-scale eddies develop vigorously as internal variability through fluid dynamic instability of the jets. Those eddies are filtered out in the statistics shown in Figs 2 and 3 but retained for those in Fig. 4. As in satellite observations (Fig. 4a), eddy kinetic energy (EKE) in the North Pacific maximizes climatologically in the KE region east of Japan, which is overall reproduced, though slightly overestimated, in our model simulations (Fig. 3c,e,g). As also revealed in satellite observations (Fig. 4b), EKE in the KE region varies on a quasi-decadal scale depending on regimes of the KE jet^3^,^15^, which is also noticeable in the simulated variability in the KE jet path (Supplementary Fig. S1). Associated with the KE jet variability, EKE is strongly suppressed in the upstream region (west of ~147 °E) relative to its downstream during the stable KE regime, for example in the period of 2002–2004 (Fig. 4b). By contrast, EKE in the upstream region is much more enhanced in the unstable KE regime in the periods of 1996–1999 and 2005–2009. This quasi-decadal EKE variability is reproduced to some extent in the B01 integration (Fig. 4d), although the duration of the stable KE regime tends to be overestimated. Though less clear, the particular quasi-decadal EKE variability is also hinted in the two other ensemble members (Fig. 4f,h), which may suggest that the quasi-decadal variability in the zonal distribution of high eddy activity region and the stability of the KE path are, to some extent, under the influence of the external atmospheric forcing through its pace-making effect^3^,^15^. Nevertheless, Fig. 4 indicates that time evolution of eddy activity in the KE region is largely intrinsic and thus unpredictable other than the potentially deterministic component discussed above.

## Summary and Discussions

Though based on the limited number of ensemble members under limited computational resource, our ensemble experiment demonstrates that a large fraction of interannual variability in the path and speed of the KE jet and associated eddy activity as well is generated through oceanic intrinsic dynamical processes and, by nature, unpredictable even under the interannually-varying atmospheric forcing. Specifically, potential predictability of interannual variability in the KE jet speed is limited substantially by intrinsic variability, whose magnitude is comparable to that of the deterministic wind-driven interannual variability. In contrast, interannual variability in the tropical ocean can be more deterministic if forced by prescribed atmospheric variability.

As suggested by our quasi-global model simulation, limitations of potential predictability by oceanic intrinsic variability apply to all the WBCs over the global ocean and associated eddies, whose heat transport and its interannual variability exert substantial influence the overlying atmosphere on various spatio-temporal scales^6^,^7^. Dynamical characteristics of the WBCs are thus analogous to those of the extratropical tropospheric circulation that varies vigorously through internal dynamics, and their variability can be considered as just a single realization among different states that could take place. Assessment of the uncertainty of interannual variability in the WBCs is, of course, impossible solely from observations. Rather, OGCM integrations with a large number of ensemble members can provide a good measure of potential predictability of the WBCs and eddy activity, although the measure obtained can be contaminated due to imperfectness of the OGCM. In recognition of the fact that the ensemble members used in the present study are obviously too few to obtain robust statistical properties of the uncertainty in the ocean variability, similar hindcast experiments with much greater ensemble size are now underway. Furthermore, as no OGCM can be perfect, multi-model ensemble experiments are necessary for assessing model dependence of the uncertainty due to biases in simulated fields.

## Methods

We use the Modular Ocean Model 3 OGCM^27^ with substantial modifications added for its optimal performance on the vector-parallel architecture of Japan’s Earth Simulator. For example, each routine is optimally vectorized, and further details can be found in Masumoto *et al.*^28^ This Ocean model for the Earth Simulator (OFES)^28^,^29^,^30^, which includes sea ice processes^31^, covers the North Pacific basin (20 °S–68 °N, 100 °E–70 °W) with horizontal resolution of 0.1°. The model has 54 vertical levels with 5-m resolution just below the surface, and the maximum depth is 6065 m. First, we conducted a 30-year “climatological integration” forced with long-term (1979–2004) mean six-hourly atmospheric forcing from which interannual variability has been excluded. A particular flow field simulated in the 16th year of that integration was used as the initial condition for our ensemble hindcast integration (ensemble A) that is forced by six-hourly atmospheric fields taken from the Japanese 25-year Reanalysis^32^ for 1979 to 2012. For our investigation of intrinsic variability in ocean currents that are subject to the interannually varying atmospheric field, two additional ensemble members (B01 and B02) were conducted from January 1st, 1995 under the identical atmospheric forcing but with integration time steps (160 seconds) that is slightly longer than for the ensemble A (120 seconds). The difference in the time steps makes slight difference in the model numerics between ensemble A and B. The initial conditions for the B01 and B02 integrations are slightly different, as they were taken from the ensemble A for the 1st and 11th days, respectively, of January 1995.

The simulated fields of the individual ensemble members are compared with observations in Supplementary Figs S2,S3 and S4. Figure S2 indicates that the mean SSH distributions in the western North Pacific, including the KE region, are well represented in each of the ensemble members. The Kuroshio off the southern coast of Japan tends to be biased toward a meandering path in the model, though. In Fig. S2 the observed and simulated SSH values themselves are different, because of the different bases used for the SSH evaluation. SST fields in Supplementary Fig. S3 are also well reproduced in the model, although horizontal SST gradient in the subarctic frontal zone to the east of Japan (around 40N) is slightly weaker than the observational counterpart.

As the average over the three ensemble members, time-mean values and interannual variance of SSH are overall reproduced well in the model with respect to their geographical distributions and amplitudes (Supplementary Fig. S4). Still, some discrepancies are noticeable between the simulated and observed fields. In the simulated time-mean field, for example, detailed structures of the subtropical frontal zone in the western North Pacific (20–25 °N) are missing, and SSH gradients under the Intertropical Convergence Zone (around 5–10 °N) are somewhat overestimated. In addition, the SSH variance is underestimated east of the Mindanao Island and in the eastern North Pacific. Despite these discrepancies, the overall model performance seems excellent.

In addition, we analyzed output of the same OGCM but expanded for a quasi-global domain and driven by atmospheric forcing that was taken from the U.S. National Centers for Environmental Prediction/National Center for Atmospheric Research (NCEP/NCAR) reanalysis^33^. A climatological integration of the model^28^ was performed for 97 years under the climatological monthly atmospheric fields with no interannual variability, and the data for the last 16 years are used to plot Fig. 3c. We also analyze global gridded delayed-time merged SSH anomaly^34^ (SSHA) and surface geostrophic current field reference products^35^ distributed by the Archiving, Validation and Interpretation of Satellite Oceanographic data (AVISO). The current data products are available on a 1/3° Mercator projection grid, and their monthly-mean values are used in this study.

The KE jet speed in Fig. 1g–i is defined as the current velocity at the 100-m depth along its axis based on monthly-mean data. It is evaluated at each zonal grid point as the maximum current velocity within the latitudinal band of 30–40 °N.

## Additional Information

**How to cite this article**: Nonaka, M. *et al.* How potentially predictable are midlatitude ocean currents? *Sci. Rep.* **6**, 20153; doi: 10.1038/srep20153 (2016).

## Supplementary Material

Supplementary Information
