# Supplementary material for: How potentially predictable are midlatitude ocean currents?
Source: Sci Rep. 2016 Feb 1;6:20153. doi: 10.1038/srep20153 (PMC4735584; doi:10.1038/srep20153)
Supplement: Supplementary Information [file srep20153-s1.pdf]

How potentially predictable are midlatitude ocean currents?

Masami Nonaka[1*]
Yoshikazu Sasai[1]
Hideharu Sasaki[1]
Bunmei Taguchi[1]
and
Hisashi Nakamura[1, 2]

[1]Japan Agency for Marine-Earth Science and Technology, Yokohama, 236-0001, Japan
[2]Research Center for Advanced Science and Technology, University of Tokyo, Tokyo, 153-8904, Japan

Submitted to *Scientific Reports*

Corresponding author address:
Masami Nonaka
Japan Agency for Marine-Earth Science and Technology (JAMSTEC),
3173-25 Showa-machi, Kanazawa-ku, Yokohama, 236-0001 Japan
nona@jamstec.go.jp

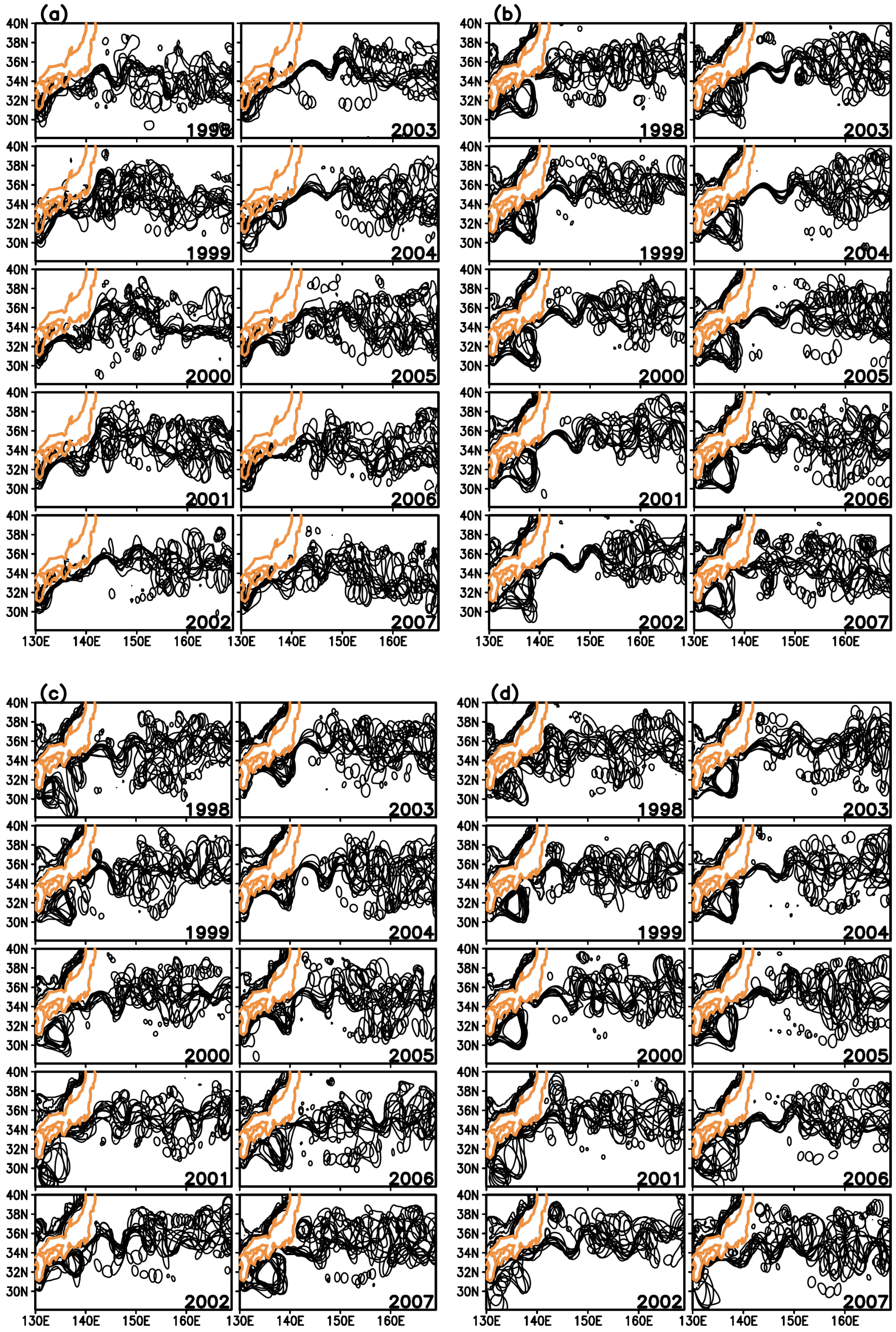

Figure S1. Stability of the Kuroshio and Kuroshio Extension path. Following Qiu and Chen (2005, 2010, 2014), yearly paths of the jet marked by a given monthly SSH contour based on satellite observations (**a**; SSH = 100 cm) and for the hindcast ensemble members B01 (**b**), B02 (**c**), and A (**d**) (SSH = –10 cm). All plots are generated with GrADS version 2.0.a8 (http://grads.iges.org/grads/head.html).

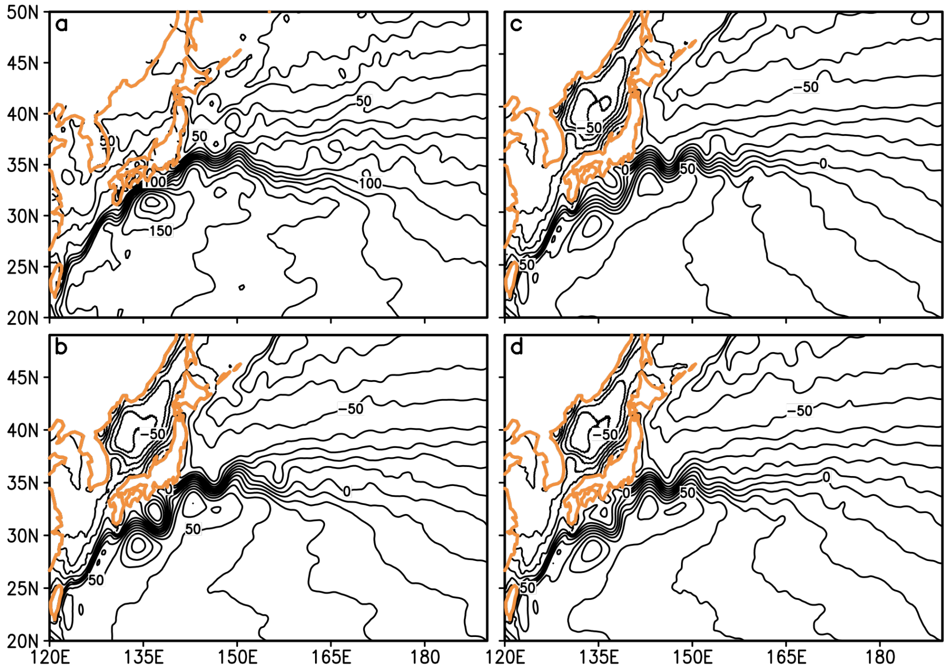

Figure S2. Long-term mean fields of sea surface height (SSH). 1995-2012 annual mean SSH field based on observations (**a**) and for the ensemble members B01 (**b**), B02 (**c**), and A (**d**). Contour intervals are 10 cm. All plots are generated with GrADS version 2.0.a8 (http://grads.iges.org/grads/head.html).

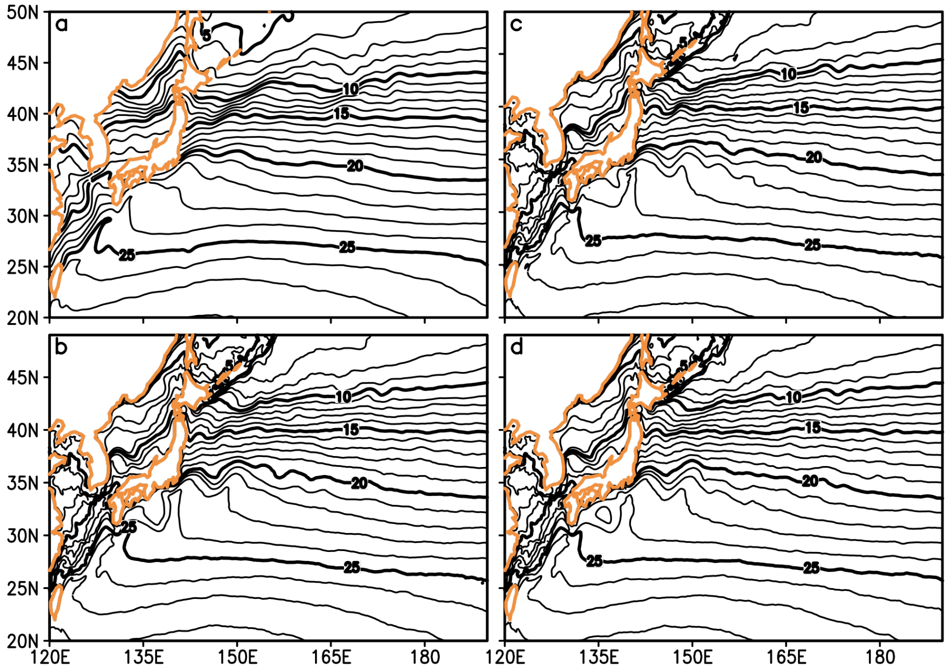

Figure S3. Long-term mean fields of sea surface temperature (SST). Same as Figure S2 but for SST field. Contour intervals are 1.0 °C. Observed SST data product of Reynolds et al. (2007) is used for panel **a**. All plots are generated with GrADS version 2.0.a8 (http://grads.iges.org/grads/head.html).

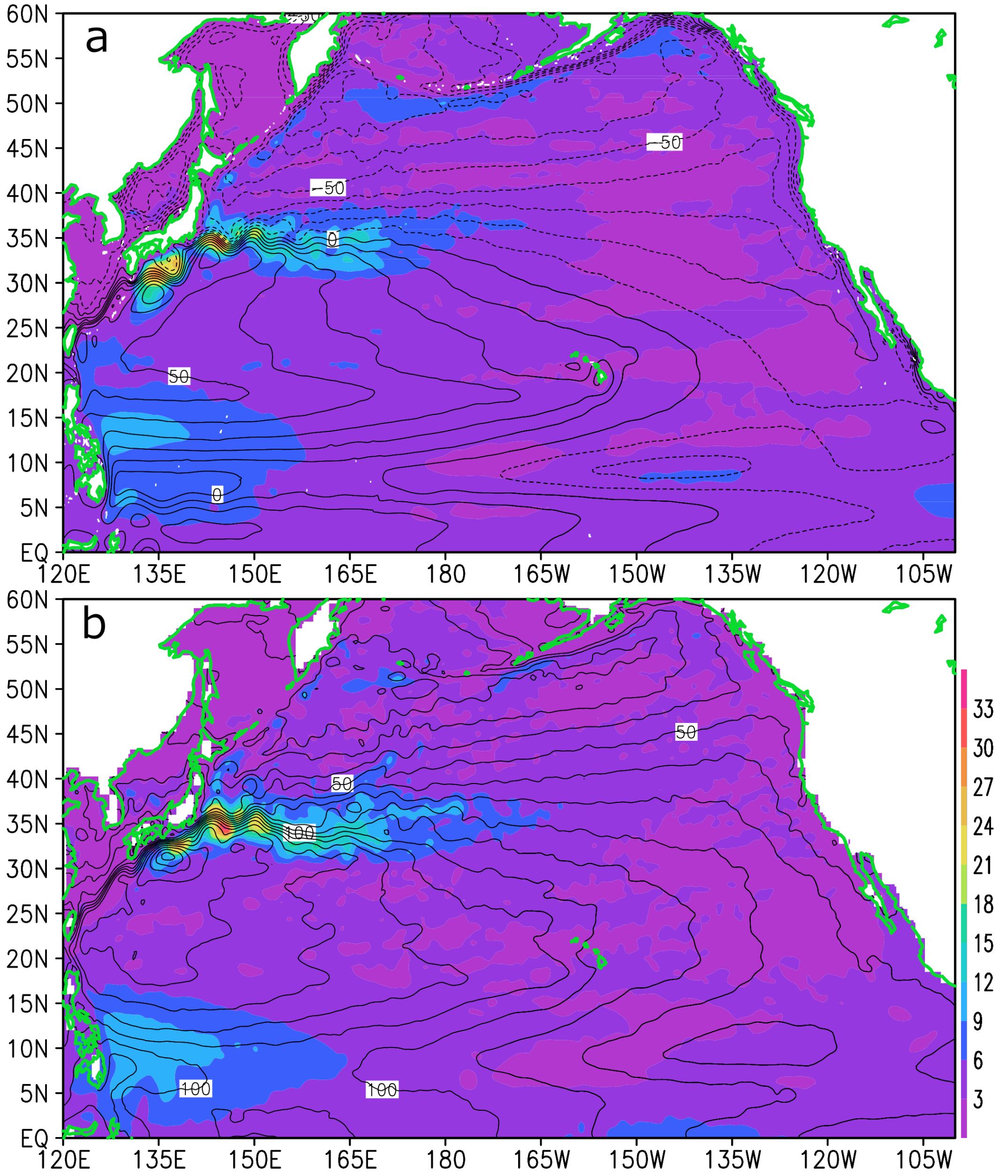

Figure S4. Maps for the ensemble mean and observed fields of sea surface height (SSH) and its standard deviations. SSH standard deviations of its 13-month running-mean time series in 1995-2012 (shades, unit is cm) and the annual mean SSH field (contours with intervals of 10 cm) in the same period for the average over the three ensemble members (**a**) and for the observations (**b**). The running mean is applied to highlight interannual variability. All plots are generated with GrADS version 2.0.a8 (http://grads.iges.org/grads/head.html).

References in Supplementary Material

1. Qiu, B., & Chen, S. Variability of the Kuroshio Extension jet, recirculation gyre and mesoscale eddies on decadal timescales. *J. Phys. Oceanogr.* **35**, 2090–2103 (2005).
2. Qiu, B., & Chen, S. Eddy-mean flow interaction in the decadally modulating Kuroshio Extension system. *Deep-Sea Res.-II* **57**, 1098-1110 (2010).
3. Qiu, B., Chen, S., Schneider, N., & Taguchi, B. A coupled decadal prediction of the dynamic state of the Kuroshio Extension system. *J. Clim.* **27**, 1751-1764 (2014).
4. Reynolds, R. W., Smith, T. M., Liu, C., Chelton, D. B., Casey, K. S., Schlax, M. G. Daily High-Resolution-Blended Analyses for Sea Surface Temperature. *J. Clim.* **20**, 5473-5496 (2007).
